# Comparison of mtDNA control region among descendant breeds of the extinct Zaupel sheep revealed haplogroup C and D in Central Europe

**DOI:** 10.1002/vms3.585

**Published:** 2021-07-22

**Authors:** András Gáspárdy, Beate Berger, Jelka Zabavnik‐Piano, Endre Kovács, Kata Annus, Petra Zenke, László Sáfár, Ákos Maróti‐Agóts

**Affiliations:** ^1^ Department of Animal Breeding Nutrition and Laboratory Animal Science University of Veterinary Medicine Budapest Hungary; ^2^ Federal Ministry of Agriculture Forestry Environment and Water Management Institute for Organic Agriculture and Biodiversity Wels‐Thalheim Austria; ^3^ Veterinary Faculty Department of Biochemistry Molecular Biology and Genetics University of Ljubljana Ljubljana Slovenia; ^4^ Hungarian Sheep and Goat Breeders’ Association Budapest Hungary

**Keywords:** breed preservation, founder sampling, haplotype diversity, maternal lineages, Zaupel sheep

## Abstract

The consideration of the descendance is indispensable in the preservation of endangered animal breeds.

The authors compared mitochondrial DNA (mtDNA) control region sequence in three descendant breeds of the extinct Zaupel sheep, firstly. Their investigation was carried out in order to prove the common origin of Waldschaf (Austria), Bovec sheep (Slovenia) and Cikta (Hungary).

A total of 118 biological samples were taken from non‐related representatives of the three breeds between 2015 and 2017. A newly designed primer pair was also used to amplify the segment (1180 bp) to be tested.

The total number of haplotypes in the whole study population was 49. The majority of which fell into haplogroup B. The significant negative value of the Fu's *Fs* statistic (*Fs* statistic = −3.296, *p* = 0.013) based on haplotype frequencies demonstrated a moderate foreign gene flow. As a novel observation haplogroups C and D appeared in Cikta and Bovec sheep, respectively. The Tajima D‐test value in the entire study population was −0.914 (*p* > 0.10), meaning that the separation of the three descendant breeds did not cause genetic drift, these are collectively in genetic equilibrium.

The genetic information confirmed the common origin of the breeds known from the breed history.

## Graphical abstract

This manuscript deals with significant questions of the common root of Zaupel sheep's descendant breeds and investigates these breeds based on mtDNA control region using a novel approach called founder sampling method. Genetic information confirmed the origin of the breeds known from the breed history separation of the three descendant breeds did not cause genetic drift, these are collectively in genetic equilibrium. At the same time, pedigree analysis that determines sampling leads to a more realistic diversity of results.

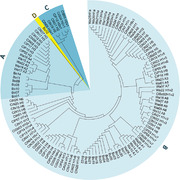

## 1 INTRODUCTION

The origin of the Zaupel sheep (Zaupelschaf) can be traced back to the Neolithic peat sheep (*Ovis aries palustris*; Mason, 1967), which, as a former companion of the Indo‐Germanic tribes, populated areas stretching from the Balkans, through the Alps, to the Pyrenees (Bohm, 1878). Excavations show that in the European Early Middle Ages, the fallow sheep existed as its only successor throughout Europe (Bökönyi, 1958). This was then split into several types per region as a result of selection and improvement. The ancient German fallow sheep existed in five variants (Golf, 1939), one of which was the prolific, mixed‐wool Zaupel sheep (*O. aries Germanicus rusticus*; Heyne, 1916). With the arrival of larger, better muscled, yet finer‐wooled foreign sheep, the fate of the Zaupel sheep was sealed from the middle of the 15th century (Adlung, 1912). The breed was permanently lost by the middle of the 20th century.

Of the extinct but historically important Zaupel sheep, four independent, still existing gene reserve breeds originated in Central Europe (Seibold, 1990). Today, as transboundary breeds, there are the Bavarian Waldschaf in Germany (its versions are Waldschaf in Austria and the Sumavska in the Czech Republic), the Alpine Steinschaf in Austria (versions of this are Steinschaf in Bavaria, and Bovec sheep or Bovska in Slovenia) and Mountain Sheep (Bergschaf; also with brown colour variations living as Engadiner Fuchsschaf in Switzerland, and as Braunes Bergschaf in Germany and Austria). The fourth Zaupel sheep descendant who came to Hungary with Swabian immigrants of the 18th century is the Cikta (Koppány, 2000).

It is likely that each descendant breed of Zaupel sheep would also have become extinct decades ago if the interest in conserving native farm animals had not been strengthened.

Mitochondrial DNA (mtDNA) is an extra nuclear, non‐recombining material of inheritance in the mitochondria. This circular genome is mainly used in evolutionary research, in the phylogenetic study of mammalian species and populations by maternal origin (e.g., Tapio et al., 2006), but also in forensic genetics (e.g., Zenke et al., 2017).

mtDNA, which, like in humans, is about 16,500 base pairs in sheep, is also used to study the history of domestication, and in this respect we can get an idea of the relative history of the breeds. According to the sheep mtDNA control region (CR), there are several major lineages (haplogroups A, B, C, D, E, F and G; later two were extinct), of which group B is found mainly in European breeds (Dymova et al., 2017).

Research conducted in the neighbouring countries of the Balkan Peninsula has dealt with the classification of domestic sheep breeds into haplogroups. For example, Cinkulov et al. (2008) found that in the West Balkan Pramenka sheep, the B haplogroup is predominant, but in addition, haplogroup A occurred in traces. The investigation of Ferencakovic et al. (2013) pointed out that the East Adriatic sheep breeds have a homogenous maternal origin of haplogroup B, while Dubrovnik Ruda sheep and Istrian sheep differentiate from them with few individuals displaying haplogroup A. Three Romanian breeds (Turcana, Tsigai and Black Head Ruda) were clustered by Dudu et al. (2016) exclusively in haplogroup B. In the fourth breed of their work, the Romanian Racka which is a variant of Turcana the haplogroup A was also detectable, but none of the Racka haplotypes were associated with C, D or E lineages.

Within Europe haplogroup C has been found, so far, only on the Iberian Peninsula (in Portugal: Pereira et al., 2006 and in Spain: Pedrosa et al., 2007) and in the southern countries of the Balkan Peninsula (in Albania and Greece: Pariset et al., 2011). The haplotype C, also identified in Egyptian breeds (Othman et al., 2015) is also in agreement with the assumption of early spread in sheep. The Iberian Peninsula was a springboard for the appearance of Asia Minor sheep on the European stage since it was connected directly to the North Africa and Asia Minor through the Mediterranean Sea.

The European spread of sheep could have occurred on a number of highly probable routes, partly along the Eastern European plain and along the Danube Valley, through the Carpathian Basin (Schmölcke et al., 2018).

Haplogroups D and E are the least frequent and have only been identified in samples from Turkey and the Caucasus (Meadows et al., 2007; Tapio et al., 2006). In the study of Liu et al. (2016) on 15 indigenous Tibetan sheep populations, the frequency of the rarest haplogroup D was less than 0.2%. Fossils in Anatolia showed a 3% presence of haplogroup E in the Bronze Age (Demirci et al., 2013).

Dymova et al. (2017) carried out archaeological mitochondrial DNA CR analysis based on about 4000‐1000 years old sheep bone remains in Altai and found all the five recent haplogroups including lineages D and E. That richness of diversity led them to conclude that the Altai region had been a migratory area for many sheep and peoples in the past.

Haplogroup E was detected in Iran in 10 Iranian native breeds (Rafia & Tarang, 2016).

Kirikci et al. (2018) stated the Karayaka breed from Northern Anatolia cannot be categorized as a genetically homogeneous population, but even has four different haplogroups (A, B, C and E).

Our hypothesis was that, with modern genetic knowledge, we would be able to show common roots and connections in breeds considered to be of common origin. If this is true, then statistical processing of the data does not justify a significant genetic difference between descendant breeds of Zaupel. In this sense, we surveyed the diversity of maternal genetic background in the following three breeds: Waldschaf, Bovec sheep and Cikta based on the sequence order of the mutable CR of the mitochondrion. With our results we wanted to prove genetically the common origin of the related varieties known from their history, and on the other hand to create a basis for the further maintenance of the genetic characteristics of the representatives in the Zaupel breed group.

## 2 MATERIALS AND METHODS

To avoid the random sampling ancestries provided information. Biological samples were taken from non‐closely related individuals in Waldschaf (Austria, n = 27) and Bovec sheep (Slovenia, n = 21) breeds using three‐generation pedigrees. In Bovec sheep, blood samples were collected from potential breeding rams included in the regular prion genotyping program, determined by order of the Ministry of Agriculture, Forestry and Food of the Republic of Slovenia. All rams were offspring of different ewes. In Cikta sheep (Hungary, n = 70), the so‐called ‘founder sampling’ was used (Maróti‐Agóts et al., 2008). In this case, sample collection was preceded by processing of the entire pedigree (Posta et al., 2019). In this breed, we collected samples from the living female representatives of the oldest families (with 4‐5‐6 ancestral generations) according to the pedigree found in the flock‐book which was re‐established in 2000. All biological samples were taken between 2015 and 2017.

DNA was isolated using the GenElute Blood Genomic DNA Kit (Sigma‐Aldrich) according to the manufacturer's instructions. The 25 μL PCR reaction mixture prepared for each sample contained 2.5 μL dNTP (10 mM), 2.5 μL 10× PCR buffer, 1.5 μL MgCl_2_ (25 mM), 2 μL primer (10μM), 1 μL BSA (20 mg/mL), 0.4 μL Taq polymerase (5 U/μL) (ThermoFisher Scientific) and 10 ng DNA template and PCR grade water to volume. Two primer pairs were used to amplify the segment to be tested: newly designed primers (Primer Designer 4.0 software) for the beginning of the region, and with a second pair as described by Hiendleder et al. (1998). First, the following amplification protocol was used for the newly designed primers (MtOA_F15400 5′‐ACACCCAAAGCTGAAGTTCTAC‐3′ and MtOA_R16087 5′‐GTTGGTTTCACGCGGCATGGT‐3′): an initial cycle of 94°C for 20 s, followed by 34 cycles of 94°C for 30 s, 62°C for 30 s, 72°C for 45 s and a final extension step 72°C for 7 min. The expected PCR product size was 688 bp. The second amplification protocol was used as follows (MtOA_F15983 5′‐AACTGCTTGACCGTACATAGTA‐3′ and MtOA_R592 5′‐AGAAGGGTATAAAGCACCGCC‐3′): an initial cycle of 94°C for 30 s, 6 cycles of 30 s at 94°C, 30 s at 54°C, 45 s at 72°C, 6 cycles of 30 s at 94°C, 30 s at 53°C, 45 s at 72°C, 21 cycles of 30 s at 94°C, 30 s at 52°C, 45 s at 72°C; and a final extension step 72°C for 7 min. The expected PCR product size was 1246 bp. A programmable Thermal Cycler 2720 PCR equipment (Applied Biosystem) was used to amplify the DNA sequence. PCR products were purified with the SIGMA GenElute™ PCR Clean Up Kit (Sigma‐Aldrich) according to the protocol.

BigDye® Terminator version 3.1 Cycle Sequencing Kit (ThermoFisher Scientific) was used for sequencing reaction in the manner recommended by the manufacturer. For sequence detection, an ABI Prism 3130XL Genetic Analyzer (Applied Biosystems) was applied, according to manufacturer's guidelines. Sequence data were analysed using Sequencing Analysis Software 5.1 (Applied Biosystems) and aligned by Sequencher™ 4.1.2 software (Gene Codes Corp).

The length of aligned and trimmed CR sequences was 1180 bp long and corresponded to positions 15,437‐16,616 on the reference sequence (AF010406; Hiendleder et al., 1998).

Mutations were evaluated using the test proposed by Fu and Li (1993) first, and then, we used the Tajima D‐test, developed by Tajima (1989) as a method for population genetic evaluation, to analyse the detected sequence mutations.

Using DNAsp version 6.0 software (Rozas et al., 2017), the number of polymorphic sites in the entire assay sample was determined and the mean nucleotide difference within and between groups was calculated. The resulting sequences were arranged with MEGAX (Kumar et al., 2018), aligned, and the related dendrogram was drawn.

The corrected number of base substitutions within the sequences was determined by the method of Jukes and Cantor (1969) and Jukes (1990).

The distribution of haplotypes by descendent breeds was plotted using Network 10.2.00 software (fluxus‐engineering.com; Bandelt et al., 1999). The sorting of the samples into haplogroups was performed by comparing our obtained sequences with gene bank reference ones (A‐HM236174, B‐HM236176, C‐HM236178, D‐HM236180, E‐HM236182 Meadows et al., 2005; O. musimon Mouflon HM236184, O. ammon Argali HM236188, O. vignei Urial HM236186 Hiendleder et al., 2002).

All 118 novel sequences used in this study were deposited in the GenBank public database under accession numbers MW427961‐MW428078.

## 3 RESULTS

In the CR region of the whole study sample, the number of monomorphic base sites was 1048, while that of the polymorphic base sites (mutations) was 131. For the latter, 13 single (singleton site positions: 140 286 312 462 499 632 696 913 962 1093 1145 1148 1183) and 118 parsimony mutations occurring in more individuals were found.

Table 1 shows the number of individuals sampled and polymorphic positions per breeds. It can be seen that the number of mutations observed shows a positive relationship with the number of individuals included in the study. The average number of mutations per individual reveals that the Waldschaf and Bovec sheep samples show significantly greater relative diversity than the Cikta sample.

**TABLE 1:** Sample size and number of polymorphic positions

Parameter	Waldschaf	Bovec sheep	Cikta	Total
No. of individuals/sequences	27	21	70	118
No. of parsimony sites/mutations	68	52	108	131
Avg. no. of mutations per individual	2.52	2.48	1.54	1.11

The total number of haplotypes in the whole study population was 49.

**FIGURE 1:**
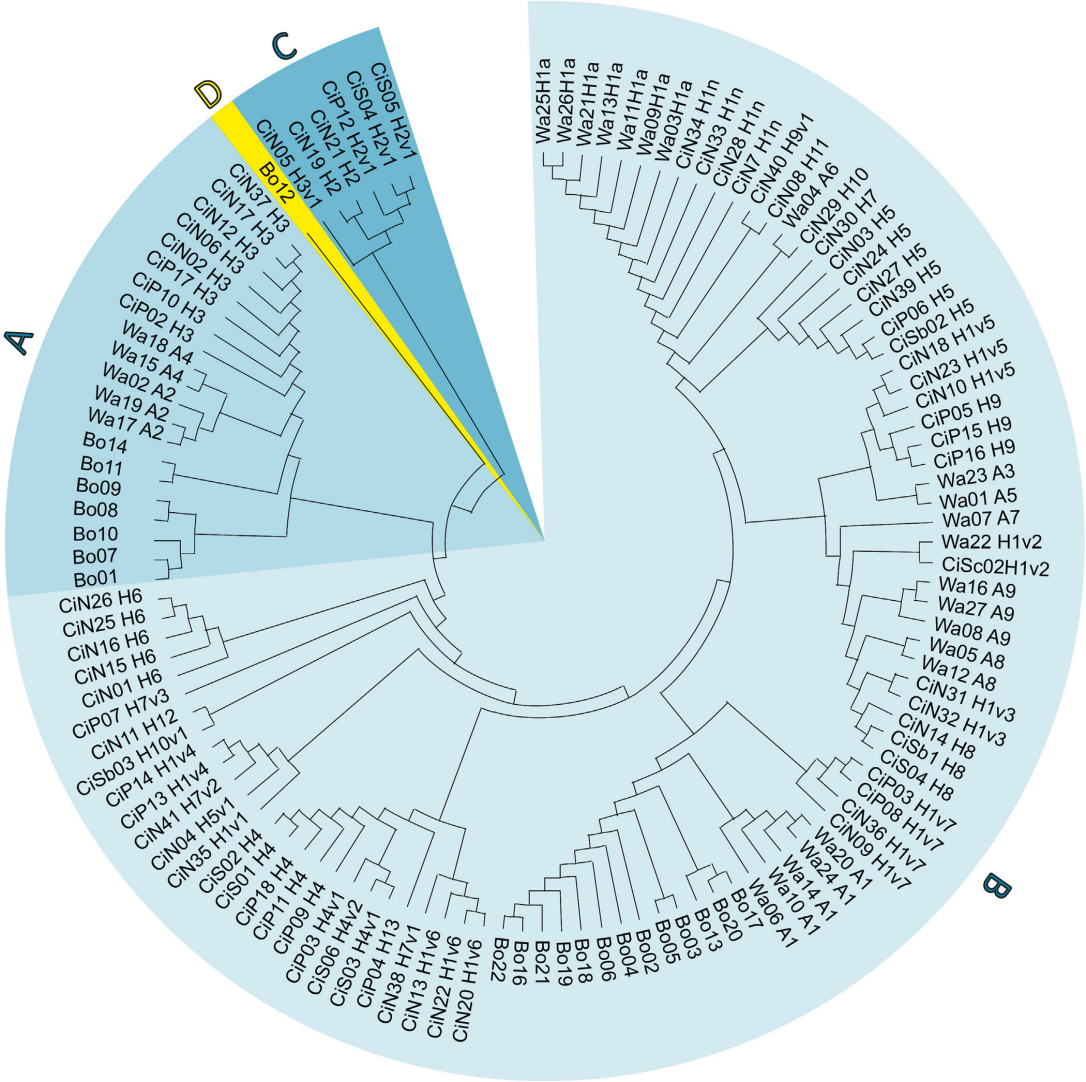
The distribution of the three related breeds according to CR haplotype and haplogroup Legend: the first two digits of the individual code refer to the breeds (Wa, Waldschaf; Bo, Bovec; and Ci, Cikta), the capital letters indicate the haplogroups, while the colours associated with the letters indicate that which individual belongs to the given haplogroup

The haplogroup and haplotype distribution of the studied breeds is shown in Figure 1. The number of haplogroups identified was four. The most populous of the haplogroups was B, followed by A, with 35, and 10 haplotypes, respectively. The dendrogram illustrates convincing the separation of haplogroups D and C from these, which include one and three haplotypes represented by one Bovec sheep and six Cikta individuals, respectively.

The comparison of related breeds is reported in Table 2 for average number of nucleotide differences (*k*) and average nucleotide diversity (π). The average nucleotide diversity (π) is the number of different nucleotides at a given base site of two randomly selected mtDNAs, which in turn determines the genetic diversity of the stock. It can be seen that the Cikta is characterized by the greatest diversity, with the highest number of average nucleotide differences (21.251) and average nucleotide diversity (18.02 * 10^−3^). This breed is followed by Waldschaf and then Bovec sheep with the least diversity.

**TABLE 2:** Values of *k* and π according to related breeds

Parameter	Waldschaf	Bovec sheep	Cikta
Average number of nucleotide differences, *k*	18.661	17.152	21.251
Average nucleotide diversity, π	15.83 * 10^−3^	14.54 * 10^−3^	18.02 * 10^−3^

Table 3 reports the values of the average number of nucleotide variations (*k*) and the average nucleotide diversity (π) in comparison between breeds. In the comparison of Waldschaf and Bovec sheep, the average number of nucleotide differences was 18.773. Here, the corrected number of base substitutions calculated according to Jukes and Cantor (Dxy (JC)) was 16.77 * 10^−3^ with a standard deviation (SD) of 2.86 * 10^−3^. The average number of nucleotide differences between Waldschaf and Cikta was 20.806, while the corrected number of base substitutions (Dxy (JC)) according to Jukes and Cantor was 17.84 * 10^−3^ with a SD of 2.37 * 10^−3^. In the third comparison (Bovec sheep and Cikta), the average number of nucleotide differences was 21,383, the Jukes and Cantor (Dxy (JC)) value with SD of 19.18 * 10^−3^ and 2.36 * 10^−3^, respectively.

**TABLE 3:** Pairwise averages of *k* and π for the three related breeds

Parameter	Waldschaf and Bovec sheep	Waldschaf and Cikta	Bovec sheep and Cikta
Average number of nucleotide differences, *k*	18.773	20.806	21.383
Average nucleotide diversity, π	15.91 * 10^−3^	17.65 * 10^−3^	18.14 * 10^−3^
Overlapping mutations, *n* and %	43, 36%	51, 29%	42, 26%

The ratio of shared mutations between breeds occurred in inverse proportion to the values of *k* and π.

According to the Tajima test performed in the entire study population, the average number of pairwise nucleotide differences (*k*) was 20.880 and the average nucleotide diversity (π) was 17.71 * 10^−3^. The Tajima D‐test value was −0.914, not statistically significant (*p* > 0.10). A significant positive value would be an indicator of genetic narrowing (bottle neck effect) or disintegration into subpopulations of a given population. Meanwhile, a significant negative value would be an indicator of selection that intends to get rid of an undesirable gene or, suitably, demographic expansion.

The Fu and Li's D* and F* tests performed on the entire pool yielded the non‐significant values of 1.217 (*p* > 0.10) and 0.562 (*p* > 0.10), respectively. Furthermore, the Fu and Li's test for haplotype diversity (Hd) gave 0.973 with a SD 5 * 10^−3^. In contrast, the Fu's *Fs* statistic resulted in a significant value of −3.296 (*p* = 0.013).

**FIGURE 2:**
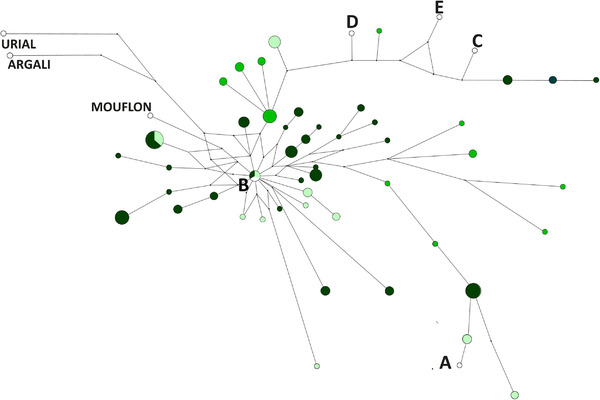
Connections between sample CR haplotypes and reference CR haplogroups by median‐joining network Legend: Waldschaf samples–light green, Bovec sheep samples–green, Cikta samples–dark green. Different white coloured spots with denomination are reference samples (A‐HM236174, B‐HM236176, C‐HM236178, D‐HM236180, E‐HM236182 Meadows et al., 2005; *O. musimon Mouflon* HM236184, *O. ammon Argali* HM236188, *O.vignei Urial* HM236186 Hiendleder et al., 2002)

The network of the connections between the found CR haplotypes and the reference CR haplogroups calculated by the median‐joining method clearly shows that the haplotypes are mainly located around the reference haplogroup B (Figure 2).

## 4 DISCUSSION

In the Tsigai, another Hungarian native sheep breed (81 individuals), 98 variable base sites were found in the CR region by Annus et al. (2015), of which 47 singleton and 51 parsimony mutations were detected. From these, it can be deduced that several related breeds show an overall more diverse genetic composition than a single breed, for example, the Tsigai which is also gene reserve breed.

The average nucleotide diversity of both breeds Gyimesi Racka and Turcana was lower (both 5 * 10^−3^) than that of all breeds currently studied, and the Tajima D‐values of both breeds were statistically proven to be in the minus range (Gyimesi Racka −1.734 and Turcana −1.814) in the evaluation of Kusza et al. (2015).

In the variant of Hungarian Racka bred in Romania Dudu et al. (2016) found similar values for Hd = 0.958 and SD = 9 * 10^−3^ taking into account the CR and cytochrome b gene together. The more frequently mutated CR, not just the longer cytochrome b sequence, may also have contributed to the relatively high values of that single breed variant.

Wassmuth et al. (2002) found that all individuals of Steinschaf (including Bovec sheep) belong to haplogroup B. However, in the Waldschaf, haplogroup A was found in more than half of the animals. It shows that not all Zaupel sheep descendants can be traced back to the same domesticated ancestor (peat sheep or *O. aries palustris*). Rather, the present study confirms that Waldschaf is also likely to be members of haplogroup B.

Among 11 Austrian sheep breeds, the shortest genetic distance was proven by Baumung et al. (2006) between Alpines Steinschaf and Waldschaf based on microsatellites, nuclear information.

The Copper Age sheep, which, according to Olivieri et al. (2012), also belongs to haplogroup B, is the possible link between the peat sheep and its distant ancestor, a certain Asiatic sub‐species of Ovis ammon.

The common genetic origin confirming the breed history may be the reason for the similarity of the three studied breeds.

The complete identity between the breeds is supported by the value of the Tajima D‐test (−0.914, *p* > 0.10) and the values of the Fu and Li's D* and F* tests (1.217, *p* > 0.10 and 0.562, *p* > 0.10, respectively). According to these values there is a limited difference in number of polymorphic sites and the mean number of nucleotide pairwise differences among the breeds. These results indicate that the community of Zaupel descendants studied is in a genetic equilibrium position, deviation from this does not endanger it, and there is no force supporting population demographic expansion.

Our joint population of Zaupel sheep descendants shows significant negative value of Fu's *Fs* statistic (−3.296, *p* = 0.013), which demonstrates foreign gene flow after a spatial expansion. The value of *Fs* statistic can be considered as low in our case, since in the clusters of other similar sheep research, it was decreased to a greater extent within the negative range with a much lower probability of error, for example, −7.48 (*p* = 0.001, Liu et al.. 2016), −10.88 (*p* < 0.001, Guo et al., 2005) and −76.28 (*p* < 0.001, Sulaiman et al., 2011). We conclude that the demographic history of the breeds differs slightly from each other, which is a sign of a diverse maternal background, that is, involvement of ewes with other genetic backgrounds in the breed. From the development of our domestic animal breeds, we have experienced that the use of the improver sires on the local dam population for several generations (up‐grading) creates a new foreign breed in a given area.

In our processing, we distinguished four haplogroups. The haplogroup B (typical for European sheep domesticated in Near East) prevailed in all breeds (Waldschaf, Bovec sheep and Cikta) with 81, 62 and 80%, respectively. This was followed by the frequency of haplogroup A (characteristic of the Indian subcontinent, 77%; Lv et al., 2015). In contrast to the former observation (Wassmuth et al., 2002), this haplogroup was found in much less than the half of animals in the Waldschaf (19%). Bovec sheep is distinguished by a remarkable proportion of haplogroup A (33%), while this haplotype is also present in the Cikta, but less typically (11%).

The today presence of haplogroup A in Central Europe should not be surprising. Region of Hungary, which is most likely amongst the earliest continental centres of wool production, served by far with the oldest samples of sheep remains in Europe assigned to haplogroup A. Sabatini et al. (2019) speculated that the haplogroup A Bronze Age sheep (1500 B.C.) in Hungary (Százhalombatta‐Földvár) may represent evidence of new sheep tentatively introduced into Europe during the Bronze Age in order to improve productions traits.

The primordial haplogroup C was first separated during phylogenetic development from B and A. The peculiarity of the processing result is that we detected the haplogroup C in the Cikta breed (with six individuals). A 9% incidence of haplogroup C indicates an even more complex maternal background of that breed. This means that ewes have entered Hungary in the past from the closer and farther areas, either from the Asia Minor (Meadows et al., 2007), Caucasus (Tapio et al., 2006), Georgia (Kunelauri et al., 2019) and Caspian See region (Lv et al., 2015) or from Mongolia (Ganbold et al., 2019) and China (Chen et al., 2006; Guo et al., 2005) where, to the best of our knowledge, haplotype C can be found outside Europe. Additionally, Lv et al., 2015 found significantly higher frequency of haplotype C in fat‐tailed breeds than in short‐tailed breeds, and the highest level of haplotype C variability in the breeds of North China. In north‐eastern India as well, the unique maternal lineage C (in the endangered Tibetan and Bonpala breeds) was unveiled by Sharma et al. (2020) despite the predominance of haplogroup A (89%) in the 11 investigated sheep breeds of India. According to the authors these two breeds migrated to that agro‐ecological region of India with the Tibetan traders, who used them as pack animals for carrying various merchandise, and share historical genes with the sheep breeds of the high‐altitude Himalayan region of China.

The other novelty is the discovery of the very rare haplogroup D at 5% level in the Slovenian Bovec sheep breed, which thus also consists of three haplogroups. Although Mariotti et al. (2013) have detected its presence in breeds Bergamasca (17%) and Laticauda (7%) in the neighbouring Italy. In our study, the Slovenian Bovec sheep, although considered to belong to haplogroup D, had fallen between D and E, as it is 33 mutations away from GenBank D and 37 mutations separate it from GenBank E.

Sheep belonging to haplogroup C and D may have either appeared in Europe with the prehistoric man, or may have later came from Asia during migration periods. With regard to the native sheep of Hungary, the last such ‘incomplete recorded’ period can be considered to be the Ottoman Empire in the 16‐17th century. This is when, fat‐tailed sheep, such as the Karagül and Kivircik (with haplogroup C frequency of 6 and 4%, respectively; Demirci et al., 2013) may have appeared which then merged into local populations. The D haplotype found in Bovec sheep is most likely explained by the genetic acquisition of Bergamasca Alpine sheep due to its geographical proximity. Therefore, it is conceivable that the haplogroup C and D is also detectable in other native breeds in Hungary and Slovenia or other surrounding countries. Clear haplogroup E has not been detected by us or even by others in Europe.

Knowledge of pedigree data is essential for the identification of maternal lineages in mitochondrial genome examinations. For representative sampling, we consider it important to use the founder sampling method because the values of true mitochondrial diversity can be calculated in this way. Furthermore, finding a unique haplotype for rare families with a high probability is likely to be the only approach to do so. It is an interesting question whether individuals with specific haplotypes should be excluded as outliers from the maintenance of autochthonous breeds.

In addition, our work draws attention to the importance of maintaining families and within family selection. The increased emphasis on the maternal side is also justified by the fact that females are present in a higher proportion than males and remain in breeding for a longer period of time, thus, they can be a greater custodian of the implementation and maintenance of genetic diversity.

## 5 CONCLUSIONS

Our working hypothesis, which was based on the breed histories, was verified by genetic analyses and their statistical testing. The high proportion of haplotype B and the overlapping mutations found in the three breeds, supplemented by the Tajima D‐, Fu and Li's D* and F* tests giving non‐significant values (in each *p* > 0.10), confirmed that these indeed come from a common ancestor, the Zaupel.

The Fu's *Fs* statistic based on haplotype frequencies is a control for rare allele's excess. Its significant negative value (*Fs* statistic = −3.296, *p* = 0.013) is an indicator of population expansion (genetic hitchhiking), which demonstrates foreign gene flow after a spatial expansion.

In addition to the haplogroups B and A typical of Europe, we were also able to detect the presence of haplogroups C and D in Central Europe.

## AUTHOR CONTRIBUTIONS

András Gáspárdy: Conceptualization; funding acquisition; methodology; supervision; writing‐original draft; writing‐review & editing. Beate Berger: Conceptualization; data curation; investigation. Jelka Zabavnik‐Piano: Conceptualization; formal analysis; funding acquisition; writing‐original draft. Endre Kovács: Formal analysis; investigation; resources. Kata Annus: Formal analysis; investigation; methodology; project administration. Petra Zenke: Data curation; formal analysis; investigation; project administration; software; validation. László Sáfár: Data curation; investigation; software. Ákos Maróti‐Agóts: Data curation; methodology; software; writing‐original draft

## AUTHOR CONTRIBUTIONS

András Gáspárdy conceived and supervised the investigation and had substantial inputs into the completion of manuscript with co‐supervision of Beate Berger and Jelka Zabavnik‐Piano. László Sáfár made an effort to process pedigree data. Endre Kovács and Kata Annus conducted the sample taking. Petra Zenke dealt the DNA purification and sequencing with, and Ákos Maróti‐Agóts had substantial role in statistical data analysis and created a first draft of the article.

## CONFLICT OF INTEREST

The authors declare no conflict of interest.

### PEER REVIEW

The peer review history for this article is available at https://publons.com/publon/10.1002/vms3.585

## Data Availability

The data that support the findings of this study are openly available under MW428078 in GenBank at https://www.ncbi.nlm.nih.gov.
